# Laser Application in Dentistry: Irradiation Effects of Nd:YAG 1064 nm and Diode 810 nm and 980 nm in Infected Root Canals—A Literature Overview

**DOI:** 10.1155/2016/8421656

**Published:** 2016-07-04

**Authors:** Yves Saydjari, Thorsten Kuypers, Norbert Gutknecht

**Affiliations:** ^1^Department of Conservative Dentistry, Periodontology and Preventive Dentistry, RWTH Aachen University, Pauwelsstraße 30, 52074 Aachen, Germany; ^2^Praxis für Laserzahnheilkunde Dres. Jung & Kuypers, Neusser Strasse 600, 50737 Köln, Germany

## Abstract

*Objective*. In endodontics, Nd:YAG laser (1064 nm) and diode laser (810 nm and 980 nm) devices are used to remove bacteria in infected teeth. A literature review was elaborated to compare and evaluate the advantages and disadvantages of using these lasers.* Methods*. Using combined search terms, eligible articles were retrieved from PubMed and printed journals. The initial search yielded 40 titles and 27 articles were assigned to full-text analysis. The studies were classified based upon laser source, laser energy level, duration/similarity of application, and initial and final bacterial count at a minimum of 20 prepared root canals. Part of the analysis was only reduced microorganisms and mechanically treated root canals upon preparation size of* ISO 30*. All studies were compared to evaluate the most favorable laser device for best results in endodontic therapy.* Results*. A total of 22 eligible studies were found regarding Nd:YAG laser 1064 nm. Four studies fulfilled all demanded criteria. Seven studies referring to the diode laser 980 nm were examined, although only one fulfilled all criteria. Eleven studies were found regarding the diode laser 810 nm, although only one study fulfilled all necessary criteria.* Conclusions*. Laser therapy is effective in endodontics, although a comparison of efficiency between the laser devices is not possible at present due to different study designs, materials, and equipment.

## 1. Introduction

The bacterial contamination of the root canal system in a tooth is the main factor of pulpal and periapical lesions [1]. The polymicrobial flora comprises an almost equal proportion of gram-negative and gram-positive bacteria [2, 3]. Those that are highly pathogenic like* Escherichia coli* produce toxic substances such as proteolytic enzymes or endotoxins, which affect and damage the surrounding periodontics [4, 5]. Endodontic therapy in dentistry involves decimating these bacteria.

In the past, the removal of an infected tooth was the only method of therapy success. In the 1940s, penicillin was used to treat infected teeth and periodontal tissues [6]. However, this intervention eliminated the symptoms rather than the main cause, while unnecessary antibiotic resistances were also created.

For decontamination, the infected teeth were treated by chemical-mechanical preparation to achieve a complete removal of the entire pulp tissue [7]. In addition to the mechanical treatment of the root canals, antibacterial rinsing solutions and drugs like calcium hydroxide applied into the root canal were used for the supportive decontamination. Sodium hypochlorite (NaOCl) proved to be an efficient rinsing solution [8]. A direct contact between chemical agents and microorganisms is required to gain its bactericidal effect.

None of the known chemical agents are currently able to satisfy all demanded requirements of root canal rinsing solutions [9].

**Figure 1:**
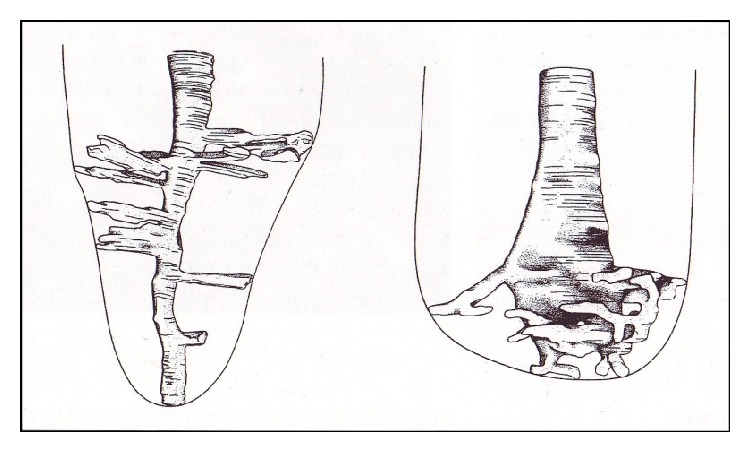
Schematic representation of the canal ramifications of teeth 13 and 24 by Blechschmidt and Meyer. A portion leads to the periodontal ligament, while another ends blindly in the dentin [20].

A lege artis primary root canal treatment lies—according to the published success—between 70 and 85% [10]. The accessory side channels leaving the main canal in the area of apex occur approximately 70% in all teeth, primarily complicating success, as shown in Figure 1.

If certain sections of a tooth are insufficiently prepared, infected tissue remains, which can lead to an exacerbation of the inflammatory process. Moreover, an effective antiseptic rinsing is not possible if the preparation size is too low [9].

The limited penetration depth (approx. 100 *μ*m) of chemical substances restricts the bacterial reduction in deeper dentin layers [11].

To remove the smear layer formed by the mechanical preparation to prevent a recolonization of the root canal system [12, 13], an extra rinsing fluid like chelate ethylenediaminetetraacetate (EDTA) or citric acid is necessary. In this case, a laser-supported root canal treatment could be an added value. Michiels et al. were able to demonstrate a significant higher reduction of reinfection of root canals after smear layer modification by the Nd-YAG laser versus an EDTA solution [14]. This result shows that the laser can also reduce the risk of leakage after root canal filling and its consequences.

In addition, adverse effects like toxicity, bad taste, and unpleasant odor of irrigation solutions have been shown in several clinical trials [15].

Spratt et al. proved in 2001 that the rinsing solution is only able to have an adequate bactericidal effect in reducing the biofilm through long exposure time [16].

Another important factor is that root canals are noncircularly sectioned yet have an oval cross section, which restricts a mechanical treatment with round instruments. A disinfecting rinsing solution combined with a laser could also provide valuable assistance to effectively remove any remaining tissue and bacteria.

Samiei et al. showed statistical differences in their in vitro study about mechanical stepback technique and laser cleaning of the root canals in teeth. The cleaning efficacy of combined laser and rotary was better than the single stepback technique [17].

Calcium hydroxide has also been proven particularly effective in root canals. This antibacterial product should remain in the root canal for at least seven days to achieve the best effect. In this context, Archilla et al. demonstrated that only a single Nd:YAG laser session is necessary to eliminate the same amount of endotoxin as calcium hydroxide is able to achieve in seven days [18]. The laser as adjunct in endodontic therapy could offer new possibilities regarding the problems described above, preventing a reinfection with its following consequences.

The laser development occurred in the 1950s, shortly after which it was used in medicine and primarily in the field of ophthalmology and dermatology. In 1971, the first CO_2_ laser was used in endodontics to seal the apical foramen [19].

The term laser (English for light amplification by stimulated emission of radiation) is an acronym describing its operating principle indeed. It acts as a light amplifier and promotes the exponential reproduction of photons due to induced emission. Each laser has various purposes in dentistry, depending upon different wavelengths.

The effects of laser irradiation in biological tissue depend on various factors [20].

*(1) Laser*

- wavelength and absorption in tissue,
- mode of operation CW (clocked, pulsed, and Q-switched),
- energy or power output (single-pulse energy/power Watt per cm^2^),
- active time (e.g., pulse duration),
- repetition rate (Hz),
- application method of the laser (contact/noncontact, focused/defocused, and rapid movements/at one point),
- time of application.

*(2) Surrounding Media*

- air,
- water,
- blood.

*(3) Tissue*

- absorption coefficient corresponding to laser wavelength,
- thermal conduction coefficient.

The laser light can be reflected on the surface* (reflexion)* or emerge after penetrating the tissue* (transmission)*. There also may be* remissions* and* diffusions* in the irradiated tissue.

The higher the absorption, the lesser the penetration depth and thermal side effects, since the energy is absorbed by the tissue absorption and its associated processes.

Laser energy can be delivered in various forms, whereby the operating mode depends on the kind of power output:

- continuous power output = continuous wave = CW,
- chopped mode,
- free running pulse,
- Q-switch mode.

Three possible theories exist for bactericidal effects of NIR laser light in the literature [21–23]:

- direct heat absorption through the bacterium itself,
- heating by absorption of the substrate in which the bacterium is located,
- photodamage effect.

The commonly used lasers in dentistry are the neodymium:YAG laser with 1064 nm, the diode laser with 810–980 nm, erbium lasers with 2940 nm/2780 nm, and the CO_2_ laser with 10600 nm.  Table 1 shows their typical fields in dentistry.

Many attempts have been made to investigate the antimicrobial potential of lasers, with numerous studies showing that the emission of laser light has a bactericidal effect in a root canal [24–29].

This literature overview provides the current state of science about Nd:YAG and diode lasers (1064 nm, 810 nm, and 980 nm) in endodontics and their action spectra in periodontal tissue with determined power settings. A comparison of these effects should evaluate a preferable laser device as support for the best results in endodontic treatments.

The Department of Restorative Dentistry at RWTH Aachen University in Germany—headed by Professor Dr. Gutknecht—has already developed a treatment protocol that could support the classic endodontic therapy concept due to the laser-specific bactericidal effect.

The proper use of the laser as an adjunct in endodontic therapy with known standards is recommended for the best clinical benefits for the patient.

## 2. Materials and Methods

To compare the variety of studies, the following criteria were selected for an adequate comparison:

- comparable operational settings of the laser device (200/300/400 microns fiber, 1.5 W, 15 pps/cw),
- similar experimental design,
- at least 20 treated root canals,
- prepared root canals to minimum ISO 30.

These parameters were chosen on account of the ability for reproduction and the actual state of knowledge by research results of the Conservative Dentistry Department, RWTH Aachen. The operational setting of 1.5 W and 15 pps showed acceptable clinical results. In these studies, the risk of possible damaging side effects was also clarified.

Some studies did not operate with contaminated teeth but rather with dentin cuts, inoculated agar plates, or animal teeth. Since these studies used at least similar parameters compared to what is mentioned above, they were also included in the general evaluation owing to the impact of the laser light on different microorganisms.

Furthermore, different variables such as the effect of the laser with respect to apical reinfection after successful root filling are listed separately or edited in Section 4, as long as they can contribute relevant information to the purpose of this review.

First, a PubMed online search was performed using specific keywords, which are listed in Table 2.

A manual search in the library of Conservative Dentistry of the RWTH Aachen was progressed, whereby the listed magazines were evaluated.

*English*

- Journal of Clinical Laser Medicine & Surgery,
- Photomedicine and Laser Surgery,
- The Journal of Oral Laser Applications,
- Lasers in Medical Science.

*German*

- Zeitschrift für Laserzahnheilkunde,
- Laserzahnmedizin Jahrbuch '11.

Most of the studies encountered in print media were also available online. The search was conducted from April 2011 until April 2016.

**Figure 2:**
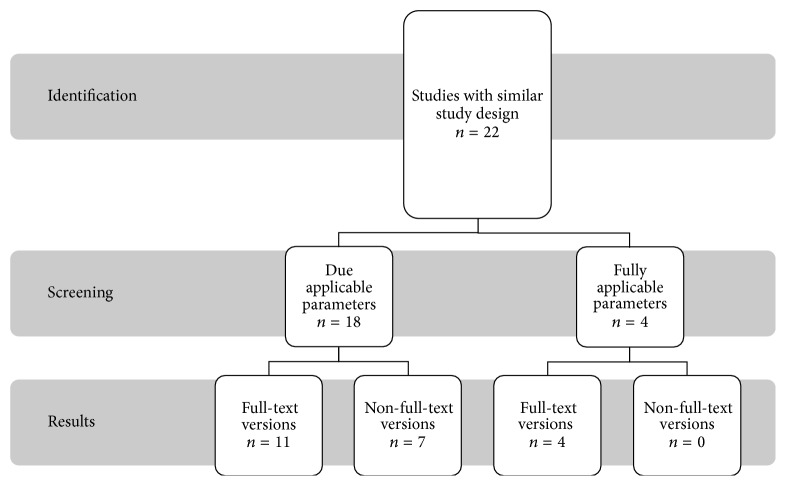
Presentation of search strategy for Nd:YAG laser 1064 nm.

For Nd:YAG laser, a total of 22 studies fulfilled the inclusion criteria for the most part and researched with rateable scientific evidence plotted in Figure 2. Four studies provide the desired requirement, while eighteen studies partly fulfilled the criteria and are listed separately.

**Figure 3:**
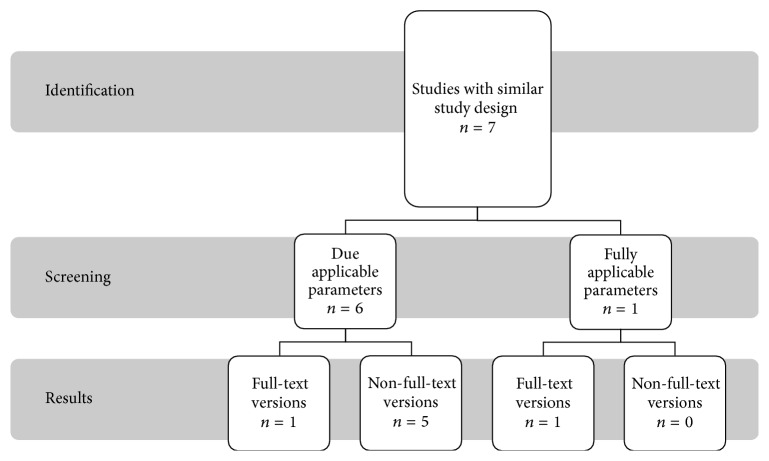
Presentation of search strategy for diode laser 980 nm.

Figure 3 shows that seven studies were evaluated for 980 nm diode laser, of which only one study fully provides the desired requirements. Six studies partly fulfilled the criteria and are listed separately.

**Figure 4:**
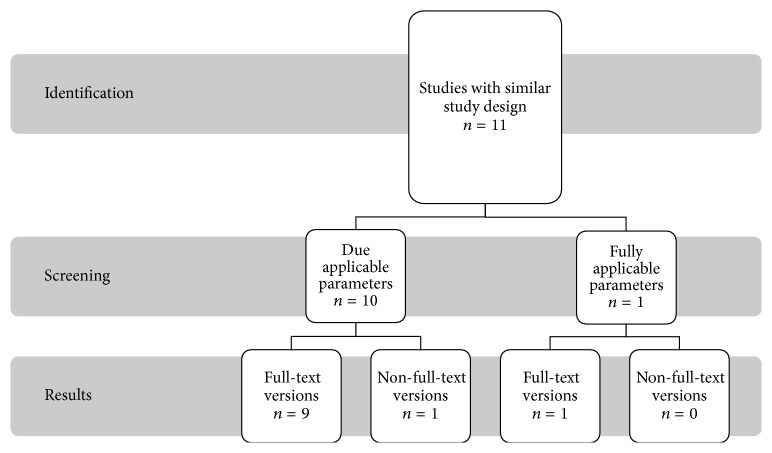
Presentation of search strategy for diode laser 810 nm.

Proceeding strictly according to the required laser settings, only one study was found for diode laser 810 nm that fully complies with the requirements detailed in Figure 4. Excluded studies contain different laser settings, and lack of information regarding the laser fiber used or a substrate was irradiated rather than teeth, but listed in Section 4 for information value.

## 3. Results

### 3.1. Studies on Nd:YAG Laser

For the Nd:YAG laser, a total of four comparable studies were found, as shown in Table 3.

Moritz et al. showed that a setting of 1.5 W for Nd:YAG laser has the best results in terms of bactericidity with less risk of thermal damage to tissue [26, 30]. They reached a bacterial reduction of 99.16% for* E. coli* and* E. faecalis*. In spite of its massive cell wall, the highly heat-resistant* E. faecalis* was sufficiently reduced [30].

Moritz et al. achieved an almost complete elimination of bacteria in their in vivo study in 1997 with the Nd:YAG laser after two radiation treatments. In 50% of cases, they reached this result after the first radiation. The maximum log kill amounted to 4.22 for* Streptococcus* and 3.33 for* Staphylococcus*. In the control group, an antibacterial solution (H_2_O_2_) was used and only one log kill of a logarithm could be achieved. In this instance, the kind of the irrigation solution should also be considered. NaOCl leads to better results in combination with H_2_O_2_. Furthermore, they also noted that a sufficient elimination of bacteria in the entire root canal can be achieved by sufficiently long exposure and adequate management of the light fiber [26].

Gutknecht et al. showed a success rate in their longitudinal study of 82% and reached a germ reduction of 84% with Nd:YAG laser up to a depth of 1000 *μ*m still [31]. In their study in 1996, Gutknecht et al. showed that between 97.91% and 99.9997% of bacteria* (E. faecalis)* were eliminated by laser radiation [32].

#### 3.1.1. Studies on Diode Laser

For each diode laser device (810 nm and 980 nm), only one study fulfilled the demanded parameters, as shown in Tables 4 and 5.

### 3.2. Diode Laser 810 nm

Beer et al. could achieve a bacterial reduction of 98.8% with the 810 nm diode laser in 2012, describing “the laser as modern state-of-the-art instrument for endodontics” [33]. Irradiation of the input cavity showed significantly better results.

### 3.3. Diode Laser 980 nm

In their study published in 2006, Schoop et al. also observed that above a setting of 1.5 W there are signs of changes in the surface and increased bactericidal effect with diode laser. The desired efficiency increases with the intensity of the laser [34].

## 4. Discussion

The comparison of the three laser systems showed that the applied formulas for calculating the actual bactericidal effect widely differ. Most studies choose different parameters of the laser device such as the intensity of radiation, exposure time, and the laser fiber used or they differ in purely practical approaches.

To investigate the actual effect of the laser on the respective microorganisms, laser fibers with a greater diameter were also used in the studies and are mentioned. In this context, clinical restrictions like heavy accessibility, strong curved root canals, or poor visibility should be eliminated. Thus, a lighter ability for reproduction could be guaranteed. These studies allow partial statements about a possible target of the selected settings to achieve the best possible bactericidal effect and are listed in Table 6.

### 4.1. Effects of Laser Light

#### 4.1.1. Thermal Effects

Across existing literature, there are relatively few studies dealing with periodontal tissue damage by overheating. In 1983, Eriksson and Albrektsson defined a heating of 47°C as critical limit for the survival of bone in rabbits [35]. Follow-up studies set a temperature increase of 10°C as the critical limit [36–39]. According to a thesis by Mazaheri in 2001 at RWTH Aachen, the maximum average temperature (10 ms interval pause, 10 ms pulse length) remains in the irradiation of root canals with the diode laser with a setting of 3 W still below the critical limit when the optical fiber is performed permanently moving coronal and apical in a circular motion in the root canal [40]. Gutknecht et al. observed a bacterial reduction in a depth of 500 microns in the teeth of cattle at a setting of 3 W cw [41]. The temperature limit is exceeded at 4 W and prolonged irradiation for 15 seconds, resulting in thermal damage.

#### 4.1.2. Power Settings

In this research, a value of 1.5 W for the diode and Nd:YAG laser has been set as an inclusion criterion. With this setting, a thermal damage is excluded within recommended handling for both laser devices and the bactericidal effects are acceptable [42]. A temperature on the root surface was observed after 45 sec. of 37°C at the recommended setting 15 pps and 1.5 W and after 90 sec. of 38°C.

In a systematic review of the current literature about the effectiveness of Nd:YAG laser on the pathogenic gram-positive bacteria* E. faecalis*, Sadik et al. showed that 1.5 W could allow an effective bacteria reduction [43].

#### 4.1.3. Effects of Laser Irradiated Root Surfaces

Gutknecht described that an application of the laser below 1 W is less important in endodontics because neither is the smear layer completely removed nor are the dentinal tubules sealed. With settings of 1.25 W–1.5 W significant changes on the root canal surface were determined. The organic material was completely removed and the surface of the inorganic substance was merged, resulting in a partial or complete occlusion of dentinal tubules [44]. This fact is to be valued positively because a reinfection is less possible with close canals.

In 2008, Klinke et al. discussed the angle between the optical fiber of the laser and the dentinal wall [45]. The laser beam hits the wall primarily at a very acute angle, depending on the mobility of the fiber in the canal, the root canal curvature, and the exit window of the laser beam from the end of the fiber. In their study, the angle between the glass fiber and dentin surface was defined as 5°. The lesser elimination of bacteria compared to other studies could result from this aspect. Further studies in terms of this angle would be interesting. The actual surface of the dentin also plays a role in terms of bactericidal effect. Darker areas cause carbonization and require a higher absorption of laser energy. The result is a local temperature increase with a bactericidal effect, albeit within no transmission of laser energy into deeper layers of dentin.

Beer et al. investigated irradiating the opening cavity of a tooth before irradiating the root canal itself, resulting in a significant higher bactericidal effect [33]. Further studies would be interesting to explore this issue in greater depth.

#### 4.1.4. Effects on Microorganisms

Pirnat et al. examined the direct effect of Nd:YAG (1064 nm) and diode laser (810 nm) on* P. gingivalis*,* E. coli*, and* E. faecalis* in 2011. They postulated two possible theories for the bactericidal effect of NIR laser light: the first refers to heating by absorption of the substrate in which the bacterium is located and the second refers to the direct absorption through the bacterium itself. In their attempt, external factors such as surrounding tissue or blood should not have an influence on the results. For this reason, they irradiated a sapphire substrate that is optically transparent for the NIR spectrum and concluded that both laser systems have a minor direct bactericidal effect on nonpigmented bacteria such as* E. coli* and* E. faecalis* [21]. However, such substrates significantly differ from the in vivo situation; for example, there is no oxygen in the bacterial microenvironment. This is necessary for the bacteria photodamage effect, although the mechanism of this degradation was not further understood [22, 23]. Future studies in this direction would be useful.

The gram-positive bacterium* E. faecalis* is more resistant in this study according to its cell wall structure compared with the gram-negative bacterium* E. coli*. The Nd:YAG laser could reduce 57% of the pigmented bacterium* P. gingivalis* and 37% could be ascertained for the diode laser. The most determining factor is believed to be the presence of the black pigment* protoporphyrin IX* in* P. gingivalis*, which absorbs the energy of the NIR light. Likewise, no growth was ascertained on the agar plates used. This fact shows that not only the bacterium itself but also its environment plays a key role for an effective endodontic laser therapy. Meire et al. irradiated bacteria inoculated agar plates* (Candida albicans, Enterococcus faecalis,* and* Propionibacterium acnes)* in a study published in 2012. The Er:YAG laser was predominant in this experiment compared to Nd:YAG laser [46]. However, the present thickness of the Er:YAG laser fiber limits an efficient transference of the light in the root canal.

The agar plates and the bacterial suspensions used in this study absorbed the laser light to a small extent. Furthermore, nonpigmented bacteria were used, which could explain the lesser effect of the Nd:YAG laser in this experiment. The different absorption of wavelengths in dentin has an effect on the depth of penetration. The Er:YAG laser had a lesser effect on the bacteria found in deeper dentinal tubules, whereas the Nd:YAG laser was significantly superior.

Meire et al. supported the statement made by Pirnat et al. that the Nd:YAG laser kills the bacteria probably by heating their environment. A comparison of studies covering the antimicrobial effect of laser light is not easy to realize because the statements about energy density or experimental conditions are often lacking. In a natural environment such as root canal wall dentin bacteria occur in a biofilm [47, 48], making them more responsive to laser light by high cell density and the presence of extracellular matrix. This fact could explain the poor action of the Nd:YAG laser on agar plates and bacterial suspensions. Different studies have shown that the bactericidal effect in the tooth is strengthened through enamel prisms and dentinal tubules as these act as a light guide [45, 49, 50]. However, additional in vivo studies are needed.

Meire et al. suppose that blood or blood products in a natural environment could lead to a raised number of* porphyrins* and* melanin pigments* in the bacteria in which the bactericidal effect is improved by Nd:YAG laser. Another interesting aspect is the dentin, which was examined more closely in a study in 1997 [51]. Carious dentin absorbs 1064 nm more wavelength in comparison to healthy dentin, which increases the desired bactericidal effect.

Hardee et al. achieved a bacterial reduction of 99% of the test bacterium* Bacillus stearothermophilus* with Nd:YAG laser, in conjunction with a log kill of 2 in comparison to a log 6 population before irradiation. Usually this bacterium is not found in infected root canals. It was selected due to its high heat resistance because the bactericidal effect of Nd:YAG laser is assumed by heat [52].

The Department of Restorative Dentistry, RWTH Aachen, currently deals with the effect of ring-firing laser fibers in the root canals, which allows the laser light to not only emit in vertical direction. New possibilities concerning the bactericidal depth effect of diode lasers and Nd:YAG lasers could be achieved.

### 4.2. Nd:YAG versus Diode Laser

A direct comparison of the selected devices is currently not feasible in relation to exact similar demanded experimental setups.

In 1997, Moritz et al. described the diode laser (810 nm) and the Nd:YAG laser (1064 nm) in endodontic treatment as equally effective and they recommended further studies to evaluate the anaerobic bacteria [26].

In a study by Kanumuru and Subbaiah in 2014, the Nd:YAG laser was most effective in the elimination of* E. faecalis* compared to 980 nm and followed 810 nm diode laser [53].

Due to the accumulation of different aggressive and resistant bacteria in an infected root canal, the additional use of Nd:YAG and diode lasers in combination with conventional methods such as mechanical conditioning or rinsing fluids seems to hold a positive value, as can be demonstrated by this literature review.

### 4.3. Nd:YAG Laser

*Advantages*. The Nd:YAG laser has clear advantages in the depth effect compared with 810 nm and 980 nm diode laser. Far more studies about Nd:YAG can be found in the literature compared to both diode lasers in endodontics. It is effective against pigmented microorganisms.

Furthermore, it removes the smear layer in a root canal, which interferes with adequate disinfection using additional rinsing fluids. It also has a simultaneous additional bactericidal effect.

*Disadvantages*. Drawbacks include the relatively high cost and its size in comparison to the two diode lasers. They are easy to handle due to their small size and the device can be used without power supply in battery mode, which Nd:YAG laser is incapable of at present.

### 4.4. Diode Lasers 810 nm and 980 nm

Comparing the 810 nm with the 980 nm diode laser, both are equally favorable. Both are adequate funds in endodontic therapy and should be investigated in further detail. For 810 nm diode lasers, the majority of studies can be found in the literature, although the parameters are not exactly comparable.

According to a study of Kales in 1993, the diode laser determines 99% of the turnover on the whole market and is estimated at 25% by the buyers in comparison to all other laser devices [54].

#### 4.4.1. Variability of Reported Results

Sadik et al. postulated that the various investigated laser systems of the past 30 years could not be compared with a meta-analysis since the results of the studies were not presented in a standardized manner. From this perspective, it would be desirable if future studies use a solid study design with the same basic parameters, such as the diameter of laser fiber, the same practical approach to the irradiation of the teeth (number of repetitions, pauses), pulse frequency (pps), and power (W) [43].

This statement is the final testimony and prime cause because this present literature review also does not lead to any clear result in terms of effectiveness brought against the bacteria in an infected root canal compared to the three lasers. There are too many different variable facts in the studies to make a statement about the more effective wavelength or the preferable device and the data situation is contradictory. The Nd:YAG laser is more frequently evaluated, although the comparability of the different study designs is also lacking. The various studies are difficult to measure, given that different parameters, fiber strengths, or handling methods are used.

At present, a statement based upon recommended guidelines is not really possible. When properly used, it emerges that disinfection by laser can increase the endodontic success with a very low risk of damaging side effects and with acceptable durability.

A recommended standardized procedure for the individual wavelengths is suggested, although further scientific studies would be desirable. Additional in vivo studies with Nd:YAG and diode lasers in endodontics are necessary. It should be considered internationally with the same procedure including a clear treatment outline. Generally established criteria such as the same fibers (diameter), the same settings of the laser parameters (power, pulse frequency), the same trace of radiation in practical implementation, and duration are essential to conduct a comparison about the antibacterial effects of endodontic treatment between the three laser devices. This would be desirable to define an evidence-based “gold standard.”

## 5. Conclusions

In endodontics, Nd:YAG laser (1064 nm) and diode laser (810 nm and 980 nm) devices are used to remove bacteria in infected teeth. This literature overview aimed to compare and evaluate the advantages and disadvantages in using these laser devices with standardized settings.

The PubMed database was searched using precise keywords between April 2011 and April 2016. Likewise, print media from the Library of RWTH Aachen University were examined.

A total of 22 eligible studies were found regarding Nd:YAG laser 1064 nm. Four studies fulfilled all demanded criteria in this review for this laser device. Seven studies referring to the diode laser 980 nm were examined, although only one fulfilled all criteria. Eleven studies were found regarding the diode laser 810 nm, but also only one study could fulfill all necessary criteria.

The analysis of the selected studies showed that all three laser systems are able to successfully decimate bacteria that are present in infected teeth. Pigmented bacteria are efficiently better removed by the Nd:YAG laser. Moreover, in deeper dentin layers, Nd:YAG laser showed better results. Concerning handiness, size, and purchase price, the diode laser is preferable.

In summary, a direct comparison cannot be made between the selected laser devices due to different study designs, materials, and equipment. Prospective randomized trials are needed to further verify which laser system is to be preferred for the best results in endodontic therapy and evaluate an evidence-based and international guideline.

## Figures and Tables

**Table 1:** Lasers in dentistry.

Laser device	Use in dentistry	Wavelength
Neodymium:YAG laser (Nd:YAG laser)	Surgery, endodontics, and periodontics	1064 nm
Erbium:YAG laser (Er:YAG laser), erbium, and chromium:YSGG laser (Er, CR:YSGG laser)	Surgery, endodontics, and cavity preparation	2940 nm, 2780 nm
Diode laser	Surgery, endodontics, and periodontics	810–980 nm
CO_2_ laser	Surgery	10600 nm

**Table 2:** Keywords used to research and their number of results on the website http://www.ncbi.nlm.nih.gov/pubmed.

Search keyword	Results
Laser in dentistry	6688
Laser, endodontics	795
Diode laser, in dentistry	614
Nd:YAG-laser, in dentistry	532
Nd:YAG-laser, root canal	160
Nd:YAG-laser, endodontics	143
Diode laser, root canal	100
Diode laser, endodontics	98
Laser, root canal	37

**Table 3:** Overview of comparable studies for Nd:YAG 1064 nm.

Year of publication	First author	Study design	Title
1999	Moritz [30]	In vitro	The bactericidal effect of Nd:YAG, Ho:YAG, and Er:YAG laser irradiation in the root canal: an in vitro comparison
1997	Moritz [26]	In vivo	Nd:YAG laser irradiation of infected root canals in combination with microbiological examinations
1996	Gutknecht [31]	In vivo	Long-term clinical evaluation of endodontically treated teeth by Nd:YAG lasers
1996	Gutknecht [32]	In vitro	Bactericidal effect of the Nd:YAG laser in in vitro root canals

**Table 4:** One study for the diode laser 810 nm matches all demanded criteria.

Year of publication	First author	Study design	Title
2012	Beer [33]	Extracted teeth	Comparison of two diode lasers on bactericidity in root canals—an in vitro study

**Table 5:** One study for the diode laser 980 nm matches all demanded criteria.

Year of publication	First author	Study design	Title
2006	Schoop [34]	Dentin cuts	Innovative wavelengths in endodontic treatment

**Table 6:** Listing of additional mentioned studies with different parameters as they occur in the text.

Year of publication	First author	Title
1983	Eriksson [35]	Innovative wavelengths in endodontic treatment
1998	Farge [36]	In vitro study of a Nd:YAP laser in endodontic retreatment
1999	Lan [37]	Temperature elevation on the root surface during Nd:YAG laser irradiation in the root canal
1997	Ramsköld [38]	Thermal effects and antibacterial properties of energy levels required to sterilize stained root canals with an Nd:YAG laser
1995	Weller [39]	In vitro radicular temperatures produced by injectable thermoplasticized gutta-percha
2001	Mazaheri [40]	Temperaturentwicklung auf der wurzeloberfläche bei einer endodontischen behandlung mit einem diodenlaser
2000	Gutknecht [41]	Diode laser radiation and its bactericidal effect in root canal wall dentin
1993	Behrens [42]	Die transmission und absorption der temperatur und energie des Nd-YAG-lasers im dentin
2013	Sadik [43]	Effects of laser treatment on endodontic pathogen *Enterococcus faecalis*: a systematic review
2004	Gutknecht [44]	Irradiation of infected root canals with Nd:YAG lasers. A review
1997	Klinke [45]	Antibacterial effects of Nd:YAG laser irradiation within root canal dentin
2011	Pirnat [21]	Study of the direct bactericidal effect of Nd:YAG and diode laser parameters used in endodontics on pigmented and nonpigmented bacteria
1999	Neuman [22]	Characterization of photodamage to *Escherichia coli* in optical traps
2008	Mirsaidov [23]	Optimal optical trap for bacterial viability
2012	Meire [46]	In vitro inactivation of endodontic pathogens with Nd:YAG and Er:YAG lasers
2007	de Paz [47]	Redefining the persistent infection in root canals: possible role of biofilm communities
1985	Nair [48]	Root canal and periapical flora: a light and electron microscopy study
1997	Klinke [45]	Antibacterial effects of Nd:YAG laser irradiation within root canal dentin
1996	Odor [49]	Pattern of transmission of laser light in teeth
1995	Vaarkamp [50]	Propagation of light through human dental enamel and dentine
1997	Jalil [51]	Surface topography of enamel and dentine from primary teeth following infrared Nd-YAG laser irradiation: an in vitro study
1994	Hardee [52]	Evaluation of the antibacterial effects of intracanal Nd:YAG laser irradiation
1997	Moritz [29]	Irradiation of infected root canals with a diode laser in vivo: results of microbiological examinations
1993	Kales [54]	Review and forecast of laser markets
2014	Kanumuru [53]	Efficacy of Ca(oH)_2_ against *E. faecalis* compared with three dental lasers on root canal dentin—an in vitro study
